# Intermittent Presumptive Treatment for Malaria

**DOI:** 10.1371/journal.pmed.0020003

**Published:** 2005-01-25

**Authors:** Nicholas J White

## Abstract

A better understanding of the pharmacodynamics of intermittent presumptive treatment, says White, will guide more rational policymaking

## Teaser

A better understanding of the pharmacodynamics will guide more rational policymaking

Intermittent presumptive treatment (IPT) in pregnancy involves giving a curative treatment dose of an effective antimalarial drug at predefined intervals during pregnancy. IPT in pregnancy was first introduced in areas of high malaria transmission as a measure to reduce the adverse impact of Plasmodium falciparum malaria in pregnancy [1,2,3,4,5,6,7,8]. Later, based on trials showing that IPT could reduce anaemia in young children and also malaria episodes in infants, it was extended as a measure to reduce morbidity and mortality in the first year of life [9,10,11,12].

Antimalarial chemoprophylaxis for pregnant women living in endemic areas has been recommended for many years, but in practice has been limited to the use of chloroquine and pyrimethamine [13,14]. Unfortunately, there are few places left in the world where these drugs can still be relied upon to prevent P. falciparum malaria. There are insufficient safety data on the newer antimalarials to warrant their systematic use in pregnant women. IPT with sulphadoxine-pyrimethamine (SP) has been introduced as an alternative. Antimalarial chemoprophylaxis in young children has been shown to reduce the adverse impact of P. falciparum malaria [15,16,17], but this intervention never obtained the same endorsement as chemoprophylaxis in pregnancy.

Five randomised trials of IPT in pregnancy in East Africa have been reported [1,2,3,4,5], all with SP, all in high-transmission settings, and all done between 1992 and 1999 (Table S1). The alarming recent increase in resistance to SP in Africa confounds the cost-effectiveness assessments upon which subsequent policy recommendations for IPT in pregnancy were based [18,19].

There is no consensus on how IPT works, making planning difficult. This article argues that IPT provides mainly intermittent suppressive chemoprophylaxis (as opposed to treatment effect alone or some other magical effects which have never been specified). If this is correct dosing schedules should be individualised for each antimalarial depending on the drug's pharmacokinetic and pharmacodynamic properties. As increasing resistance to SP must seriously compromise IPT regimens based on this drug, the evaluation of available new effective antimalarials is needed urgently, in both high- and low-transmission areas.

## Pharmacokinetics

**Figure 1:**
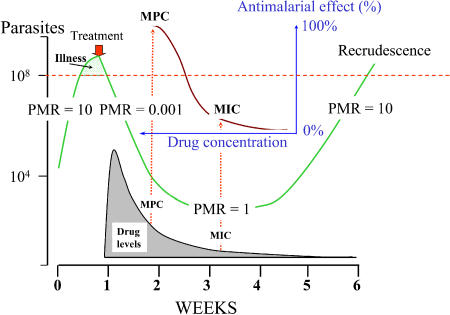
In Vivo Antimalarial Pharmacodynamics The parasite burden in an adult (vertical axis) is shown in green. After parasite burden expands to the point where it causes illness, treatment is given (red arrow), which causes a log-linear decline in parasite numbers until concentrations of the antimalarial drug (grey shading) fall below the MPC. As the antimalarial blood levels fall further, the decline in parasite burden slows until it reaches a multiplication rate of one (the antimalarial concentration at this point is the in vivo MIC). The parasite population then expands to cause a recrudescence six weeks later. The sigmoid concentration–effect relationship is shown in brown; it is depicted in the reverse direction to that normally drawn. PMR, parasite multiplication rate.

**Figure 2:**
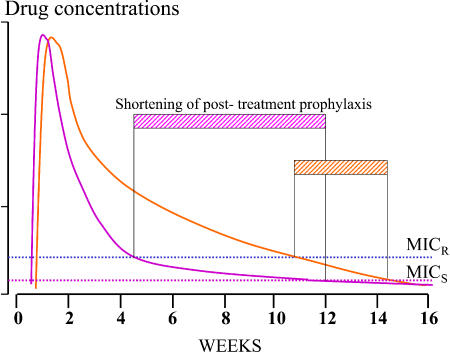
Blood Concentration Profiles of Two Antimalarials with Different Elimination Profiles The examples shown here are mefloquine (orange) and chloroquine (pink). An increase in MIC has different effects on the shortening of post-treatment suppressive prophylaxis (hatched bars). MIC_R_, MIC for resistant parasites; MIC_S_, MIC for sensitive parasites.

After a treatment dose of SP (25 mg sulfadoxine/1.25 mg pyrimethamine per kilogram body weight), plasma concentrations of pyrimethamine (half-life, 3 days) and sulfadoxine (half-life, 7 days) decline log-linearly [20,21]. The antimalarial effect depends on synergy between the two components, but the effect from one treatment dose can last as long as 60 days with fully sensitive P. falciparum [20,21]. For slowly eliminated antimalarial drugs (Table S2), the terminal elimination phase crosses the in vivo dose–response curve (Figure 1). Thus, if a full treatment dose is given, concentrations at the beginning of the terminal elimination phase exceed the minimum parasiticidal concentrations (MPCs)—the lowest concentrations that give maximum effect [22]. The exceptions to this are chloroquine (and probably piperaquine), as resistance to these drugs increases, because the elimination of chloroquine is multiexponential, and the terminal elimination phase begins at concentrations that are low by comparison with the peak concentrations after treatment (Figure 2). 

The pharmacokinetic properties of many drugs are altered in pregnancy; lower concentrations often result from an expanded volume of distribution. Strangely, despite the wide endorsement of SP IPT in pregnancy, there are no pharmacokinetic studies of sulphadoxine or pyrimethamine in pregnancy, so it is not known whether the current dosing is optimal. The absorption and disposition of many drugs are also altered in infancy, but there are very few data on antimalarial pharmacokinetics in the first year of life. For some drugs (e.g., amodiaquine) there is insufficient information for any age group.

## Pharmacodynamics

Is the benefit of IPT gained only through clearing parasites from the placenta (“treatment effect”), or is the prevention of new infections (“prophylactic effect”) an important component? If only the treatment effect is important, then how long does the beneficial effect of eradicating an asymptomatic low-density infection persist for? If it lasts until the next infection becomes patent (i.e., detectable), then rapidly eliminated drugs will provide protection only for a few days longer than the average incubation period (about two weeks). Establishment of a new placental infection (i.e., pathologically significant placental sequestration) may take longer because the placenta selects and accumulates parasites that bind to the proteoglycans chondroitin sulphate and hyaluronic acid [23]. If only the treatment effect is important, then for sustained benefit we must hypothesise that the parasites that persist asymptomatically before IPT is given are a selected subpopulation that is more pathological than the parasites that cause subsequent reinfection. This seems implausible in infancy, and even in pregnancy it seems unlikely that it would take more than ten weeks in high-transmission settings to re-establish a significant placental infection. This suggests that the prophylactic effect is important for the efficacy of IPT.

The duration of prophylactic effect is compromised particularly by resistance. For most antimalarials the duration of antimalarial effect is a simple function of the in vivo concentration–effect (dose–response) relationship and the pharmacokinetic properties of the antimalarial drug [22]. But for SP this function is more complicated, as synergy between the two components needs to be considered. The duration of synergy depends on resistance levels determined by mutations in the parasites' genes encoding dihydropteroate synthase and dihydrofolate reductase, the respective targets of sulphadoxine and pyrimethamine [20,21]. Information on this temporal pattern of reinfection following IPT in pregnancy or infancy is lacking. Such information is essential if the choice of drug and the dosing is to be rationalised.

**Figure 3:**
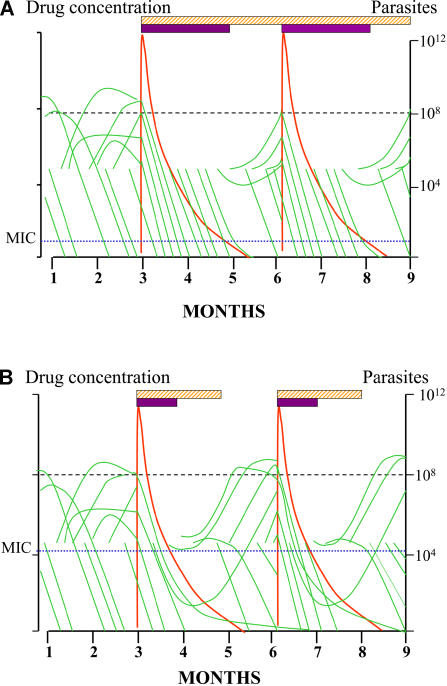
Hypothetical Parasite Burden Profiles during Pregnancy with SP IPT in a High-Transmission Setting Entomological inoculation rate is about 50 infectious bites per person per year. Note that many infections self-cure (each infection is depicted as a green line). The hatched bars represent the duration of “suppressive prophylactic activity”, and the solid bars represent the period during which parasite multiplication is suppressed (i.e., levels exceed the in vivo MIC). The horizontal dotted line at 10^8^ parasites represents the level at which malaria can be detected on a blood film. (A) represents a drug-sensitive area; (B) represents a moderately resistant area.

**Figure 4:**
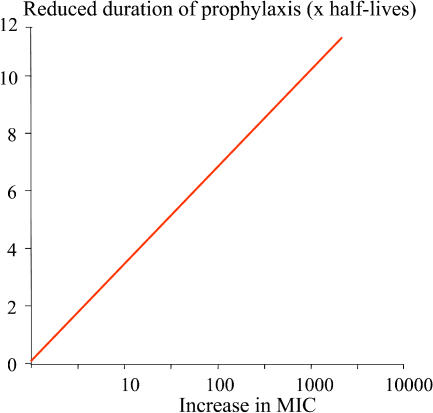
Relationship between MIC and PTP The proportional increase in malaria parasite MIC with resistance is plotted against the shortening of the duration of PTP, expressed as multiples of the terminal half-life. This applies only to drugs for which suppressive antimalarial prophylaxis occurs in the terminal elimination phase (i.e., most drugs).

Resistance is defined by a right shift in the concentration–effect relationship and results in reduced effects for any concentration below the MPC for resistant parasites [24] (see Figure 1). As the concentration of a slowly eliminated antimalarial in the blood declines, it continues to suppress the growth of newly acquired infections as they emerge from the liver. Eventually, however, concentrations fall below the minimum inhibitory concentration (MIC) for the prevalent parasites (i.e., the concentration at which the net multiplication rate is one), and parasite expansion is possible (Figure 3). It follows, then, that the duration of “post treatment prophylaxis” (PTP) (i.e., the length of time after an antimalarial treatment dose for which newly acquired infections are suppressed) is determined by the concentrations of the drugs used (determined by dose and pharmacokinetics) and the sensitivity of the prevalent parasites. The more resistant the parasites are, the shorter is the duration of PTP; for each doubling of MIC the duration of PTP is shortened by one half-life (Protocol S1). The triple dihydrofolate reductase mutants now prevalent across much of Africa have an approximate 1,000-fold reduction in pyrimethamine susceptibility, which would translate into a reduction in PTP of one month (Figure 4). As a further confounder, folic acid, which is prescribed widely in pregnancy, is a competitive antagonist of pyrimethamine. 

## Preventing Placental Pathology

In a high-transmission setting infections are acquired every few days or weeks throughout life (see Figure 3). Mortality is high in childhood, but by the time of adulthood and pregnancy, infections are largely asymptomatic—although they are often still patent (which requires a total burden of greater than 100 million parasites) [22]. Thus, immunity prevents life-threatening parasite burdens, and suppresses the pro-inflammatory response (which causes illness), but it does not prevent infection. In pregnancy this immune control is impaired in the placenta, which acts as a “privileged site” for parasite multiplication. The objective of IPT in pregnancy is to reduce or eliminate the adverse effects of malaria on maternal anaemia and birth weight, and, in addition, in a low-transmission setting, to prevent severe malaria in the mother [25,26]. How malaria produces intrauterine growth retardation is still unresolved, but in P. falciparum malaria, retardation tends to be greatest in the first pregnancy, and often occurs without maternal illness. The greater the placental parasite burden, the greater is the reduction in birth weight. “Placental malaria”—histological evidence of placental accumulation of parasitized erythrocytes or malaria pigment deposition—has often been used as an endpoint in intervention studies, although the quantitative relationship between placental malaria and reduction in birth weight remains poorly characterised.

## Preventing Malaria in Infancy

There are fewer data on the efficacy of IPT in infancy than in pregnancy (Table S3). The pharmacodynamics of IPT in infancy are probably similar to those in pregnancy, although there is no “privileged site” for parasite multiplication. Protection in the first months of life is mediated by a variety of factors, which include transplacentally acquired maternal antibody (IgG) and a relatively high haemoglobin F content in the infants' erythrocytes. After about six months of age, protection from these factors wanes, and the infant becomes much more vulnerable to malaria than the mother (because protective immunity has yet to be acquired). As delivery of antimalarials in the rural tropics is so difficult, for operational reasons IPT is currently being given to infants at the same time as the EPI immunisations (at 2, 3, and 9 months). This regimen leaves a six-month gap between the second and third administrations, which, even for fully SP-sensitive parasites, leaves four unprotected months. This is at a time when the infant is increasingly vulnerable to severe malaria. More information is needed on the duration of protection afforded by currently available antimalarial drugs when administered to healthy infants.

## Should IPT be Used in Low-Transmission Settings?

If IPT is just a simple, albeit imperfect, way of administering chemoprophylaxis, then there are also strong arguments for evaluating this approach in low-transmission settings. The adverse impact of malaria in pregnancy is greater in low-transmission than in high-transmission settings. The reduction in the birth weight of first-borne infants is similar, but extends to the second and subsequent pregnancies; treatment failure rates are higher than in non-pregnant adults [27,28,29]; and there is a significant risk of severe malaria with attendant very high mortality. In Asia and South America, where low-transmission areas predominate, P. vivax is also an important cause of low birth weight, and so useful preventative measures must also be effective against this infection [30].

Antimalarial prophylaxis has been recommended and used in low-transmission settings, but whereas chloroquine remains generally effective against P. vivax, there are no safe and effective available drugs for P. falciparum infections. Use of IPT in these areas would provide a sterner test than in a high-transmission area because there would be little or no background immunity to assist antimalarial drug efficacy.

## What Is the Correct Dose and the Correct Interval Between Doses?

The dose used in IPT is usually the full age- or weight-adjusted treatment dose derived either empirically or from dosefinding studies (usually in non-pregnant adults). The dose and dosing interval should be determined by the tolerability, absorption, distribution, and elimination kinetics of the drug used, and the in vivo MIC. Unfortunately, for the two drugs that have been used for IPT (SP and amodiaquine) there are no pharmacokinetic data in pregnant women or infants. The in vivo MIC is an important measure but it is parasite specific and difficult to assess [31,32,33,34,35]. Ideally, the interval between doses should not be more than one week longer than the time needed for plasma concentrations to fall from peak post-dose levels to the MIC value. This timing is a conservative choice as it assumes all infections are equally harmful, and it does not take into account either the delay in selecting a placenta-binding P. falciparum subpopulation in pregnancy or the delay in achieving full growth rates because of continued sub-MIC suppression. Although the MIC for drugs that are succumbing to resistance obviously varies considerably, for newer drugs such as lumefantrine or piperaquine the variance is considerably less and generalisations can be made.

## Should an Artemisinin Combination Be Used for IPT?

If IPT is simply prophylaxis, then a rapidly eliminated artemisinin component provides very little direct benefit for the additional cost and risk (although the risks are thought to be very small in the second and third trimesters of pregnancy and in infancy). The addition of an artemisinin component would accelerate parasite clearance and prevent gametocyte production, but the benefits of this in an asymptomatic pregnant woman or child are uncertain. The main benefit would be in providing protection against the emergence of de novo resistance to the slowly eliminated drug, although, because parasitaemias tend to be low, the probabilities of de novo selection are much lower than in acute symptomatic infection. But there is a genuine concern that if monotherapies are made available, then they will be used and abused, and resistance may develop.

## Discussion: The Policy Implications

Without a better understanding of the pharmacodynamic effects of IPT, it will be difficult to make rational improvements in this promising approach to malaria prevention. The most parsimonious explanation for its effectiveness is that IPT provides antimalarial prophylaxis that, if sufficiently lengthy and effective, is beneficial both to the pregnant woman and the infant. But how lengthy and how effective?

For IPT in pregnancy the only drug that has been evaluated is SP, at a time when the drug was more effective than it is today. A significant improvement in birth weight was found in only two of four randomised trials. In a large prospective observational study conducted in western Kenya, IPT was associated with an odds ratio of 0.65 (95% confidence interval, 0.45 to 0.95) for low birth weight [7]. A dose–response relationship was found, with an adjusted mean increase in birth weight of 61 g for each increment in the number of SP doses (up to three doses). So, two doses of SP did not provide maximal benefit. But given the alarming decline in SP efficacy in Africa (resulting from rapid spread of “quintuple” dihydrofolate reductase/dihydropteroate synthase mutants that are about 1,000 times less sensitive to pyrimethamine than wild-type parasites), there are grave doubts about whether the efficacy observed in these various studies would still be observed today, even if the dosing was increased. SP cure rates in children in Malawi—where IPT in pregnancy is widely used—have been consistently less than 40% for the past five years [36]. It has been suggested that asymptomatic pregnant women in high-transmission settings may have sufficient immunity to complement a failing drug—i.e., treatment responses would be better than in symptomatic children. However, if the duration of PTP is the main determinant of benefit, then this benefit is shortened progressively by increasing resistance (see Figure 4). Alternatives to SP are needed urgently.

Is IPT safe? There is no evidence to date that IPT is harmful. But the incidence of serious adverse effects when amodiaquine (agranulocytosis, 1:2,000) and SP (Stevens-Johnson syndrome,1:7,000) were used as antimalarial prophylaxis by Western travellers was so high that they are contraindicated [37]. Both drugs were associated with severe hepatitis. Single treatments are considered safer, but how much safer is not known. There are insufficient data on the safety of amodiaquine in pregnancy [38]. The closer IPT comes to continuous prophylaxis, presumably the higher the risks of serious adverse effects. The risk–benefit assessment is difficult to make, but with the current high levels of SP resistance, these important uncertainties also argue strongly for the evaluation of alternatives. Proguanil and quinine are regarded as safe, but both are eliminated very rapidly. Treatment doses of mefloquine are not well tolerated by healthy subjects, and there are safety concerns in pregnancy [39]. Serious contenders all require more than a single dose and will need urgent evaluation. These include artemether-lumefantrine, although more information is needed on safety and on the pharmacokinetics in pregnancy, and the duration of PTP provided by lumefantrine needs further assessment. It may be too short. Dihydroartemisinin-piperaquine may be the most promising candidate. It is very well tolerated, and piperaquine is slowly eliminated. Indirect evidence from the pattern of new infections following clinical trials suggests protracted suppressive activity. It has not yet been evaluated in pregnancy, so more information is needed on safety and pharmacokinetics in this context. For IPT in infancy, however, there seems every reason to evaluate this drug as soon as possible.

## Supporting Information

Protocol S1 Calculations Showing That for Each Doubling of MIC the Duration of PTP Is Shortened by One Half-Life (27 KB DOC).

Table S1 Randomised Trials of IPT in Pregnancy (31 KB DOC).

Table S2 Terminal Elimination Half-Lives of Currently Available Antimalarial Drugs (34 KB DOC).

Table S3 Randomised Trials of IPT in Infancy (27 KB DOC).

**Summary Points**

- Intermittent presumptive treatment (IPT) with sulphadoxine-pyrimethamine (SP) in pregnancy and with amodiaquine or SP in infancy has been proposed for use in areas with high levels of malaria transmission.
- The duration of post treatment prophylaxis is likely to be an important determinant of the benefit of IPT.
- Because of rapidly increasing resistance, it is very unlikely that IPT in pregnancy with SP is as effective now in east Africa as it was 5–10 years ago, when it was evaluated.
- More effective antimalarial drugs such as artemether-lumefantrine and particularly dihydroartemisinin-piperaquine should be evaluated for IPT in both low- and high-transmission settings.
- Choice of drug, dosing, and dose spacing for IPT should be based on a better understanding of pharmacokinetics and pharmacodynamics.

## Abbreviations

IPT: intermittent presumptive treatment
MIC: minimum inhibitory concentration
MPC: minimum parasiticidal concentration
PTP: post treatment prophylaxis
SP: sulphadoxine-pyrimethamine
