# Supplementary material for: Intermittent Presumptive Treatment for Malaria
Source: PLoS Med. 2005 Jan 25;2(1):e3. doi: 10.1371/journal.pmed.0020003 (PMC545196; doi:10.1371/journal.pmed.0020003)
Supplement: Protocol S1 — (27 KB DOC). [file pmed.0020003.sd001.doc]

**Protocol S1.** Calculations Showing That for Each Doubling of MIC the Duration of PTP is Shortened by One Half-life

In the terminal elimination phase the concentration (C_t_) at any time t is given by

C_t_ = C_0_ e^-kt^ ……………………………………………………………………..(1)

Where k is the first order terminal elimination rate constant and C_0_ is the concentration at the beginning of the terminal elimination phase. If the MIC is C_MIC_ then from (1)

ln C_MIC_ = lnC_0_ – kt…………………………………………………………………..(2)

k = 0.693 x t_1/2_ where t_1/2_ is the terminal elimination half-life……………….…….(3)

If there is a ***doubling*** of MIC as a result of resistance, then from (2)

ln C_MIC_ + ln 2 = lnC_0_ – kt_2_…………………………………………………….……...(4)

where C_MIC2_ is 2(C_MIC_) and t_2_ is the new interval from the beginning of the beginning of the terminal elimination phase until the end of antimalarial suppressive activity

But the time taken for plasma concentrations to fall by half (t_2_ – t) is the terminal elimination half-life, and so for each doubling of MIC the prophylactic activity is shortened by one half life.

i.e. t_2_ – t = (lnC_0_- ln C_MIC_- ln 2)/k - (lnC_0_- ln C_MIC_)/k = 0.693/k………………….(5)

so for a ***ten-fold*** increase in MIC (i.e. ln C_MIC_ + ln 10 = lnC_0_ – kt_10_ )

t_10_ – t = ln10/k = 2.303/k ……..…………………………………………….(6)

but from (3) k = 0.693/ t_1/2_ half lives

so (6) becomes = 2.303/(0.693/ t_1/2_) = **3.32** half-lives…………………………….(7)
