# Supplementary material for: Intermittent Presumptive Treatment for Malaria
Source: PLoS Med. 2005 Jan 25;2(1):e3. doi: 10.1371/journal.pmed.0020003 (PMC545196; doi:10.1371/journal.pmed.0020003)
Supplement: Table S1 — (31 KB DOC). [file pmed.0020003.st001.doc]

**Table S1. Randomised Trials of IPT in Pregnancy**

| Place | **Investigators** | **IPT Regimens** | **Effect of IPT on Anaemia** | **Effect of IPT on Placental Parasitaemia** | **Effect of IPT on Birth Weight** | **Years of Study** |
| --- | --- | --- | --- | --- | --- | --- |
| Malawi | Schultz et al. (1994) | Cq weekly, SP then Cq weekly, or SP | NE | Decreased | NS | 1992 |
| Malawi | Verhoeff et al. (1998) | SP | NS | NS | LBW decreased | 1993–1994 |
| Kenya | Parise et al. (1998) | SP, SP monthly, or case management | Decreased | Decreased | LBW; NS, but overall mean increase | 1994–1996 |
| Kenya | Shulman et al. (1999)] | SP or placebo | Decreased | NS | NE | 1996–1997 |
| Kenya | Njagi et al. (2003) | SP or placebo | Decreased | Decreased | NS | 1997–1999 |

Cq, chloroquine; LBW, low birth weight (<2,500 g); NE, not evaluated; NS, not significant (*p* > 0.05).
