# Supplementary material for: Intermittent Presumptive Treatment for Malaria
Source: PLoS Med. 2005 Jan 25;2(1):e3. doi: 10.1371/journal.pmed.0020003 (PMC545196; doi:10.1371/journal.pmed.0020003)
Supplement: Table S2 — (34 KB DOC). [file pmed.0020003.st002.doc]

**Table S2.** Terminal Elimination Half-Lives of Currently Available Antimalarial Drugs

| **Drug** | **Terminal Half-Life in Health** | **Pharmacokinetic Data in Pregnancy** | **Comments** |
| --- | --- | --- | --- |
| Artemisinin and derivatives^a^ | 1 hour | Yes | Safety in first trimester not established. Blood levels in pregnancy reduced. |
| Quinine^a^ | 11 hours | Yes | Causes hypoglycaemia in late pregnancy. Blood levels in pregnancy reduced. |
| Proguanil^a^ | 16 hours | Yes | Antimalarial activity derives mainly from metabolite cycloguanil. Conversion via CYP 2C19 impaired in pregnancy. Blood levels in pregnancy reduced. |
| Chlorproguanil | 16 hours | No | Antimalarial activity derives mainly from metabolite chlorcycloguanil. Not studied in pregnancy but probably similar to proguanil. |
| Atovaquone^a^ | 33 hours | Yes | Blood levels in pregnancy reduced. |
| Lumefantrine | 3–4 days | Yes | Desbutyl metabolite also active. |
| Halofantrine | 3–4 days | No |  |
| Pyrimethamine | 3 days | No |  |
| Sulphadoxine | 7 days | No |  |
| Amodiaquine | 1–3 weeks? | No | Desethyl metabolite accounts for nearly all antimalarial activity. |
| Piperaquine | 22 days | No |  |
| Chloroquine | 2–3 months | Yes | Desethyl metabolite also active. |

Primaquine and tetracyclines should not be used in pregnancy. There are insufficient safety data in pregnancy for all drugs with the exceptions of chloroquine, quinine, and SP.

^a^Blood levels reduced in pregnancy.
