# Supplementary material for: Intermittent Presumptive Treatment for Malaria
Source: PLoS Med. 2005 Jan 25;2(1):e3. doi: 10.1371/journal.pmed.0020003 (PMC545196; doi:10.1371/journal.pmed.0020003)
Supplement: Table S3 — (27 KB DOC). [file pmed.0020003.st003.doc]

**Table S3.** Randomised Trials of IPT in Infancy

| **Place** | **Investigators** | **IPT Regimens** | **Age Group** | **Effect of IPT on Anaemia** | **Effect of IPT on Fevers** |
| --- | --- | --- | --- | --- | --- |
| Tanzania | Schellenberg et al. (2001) | SP or placebo | Infancy | Reduced | Reduced |
| Kenya | Desai et al. (2003) | SP at 4 and 8 weeks, iron plus SP at 4 and 8 weeks, iron, or placebo | 2–36 months | Reduced | Reduced |
| Tanzania | Massaga et al. (2003) | Amodiaquine, iron, amodiaquine plus iron, or placebo | Infancy | Decreased 67% | Decreased 65% |
